# Genetic impacts on DNA methylation: research findings and future perspectives

**DOI:** 10.1186/s13059-021-02347-6

**Published:** 2021-04-30

**Authors:** Sergio Villicaña, Jordana T. Bell

**Affiliations:** Department of Twin Research and Genetic Epidemiology, St. Thomas’ Hospital, King’s College London, 3rd Floor, South Wing, Block D, London, SE1 7EH UK

**Keywords:** DNA methylation, Heritability, GWAS, Methylation quantitative trait loci, meQTL

## Abstract

Multiple recent studies highlight that genetic variants can have strong impacts on a significant proportion of the human DNA methylome. Methylation quantitative trait loci, or meQTLs, allow for the exploration of biological mechanisms that underlie complex human phenotypes, with potential insights for human disease onset and progression. In this review, we summarize recent milestones in characterizing the human genetic basis of DNA methylation variation over the last decade, including heritability findings and genome-wide identification of meQTLs. We also discuss challenges in this field and future areas of research geared to generate insights into molecular processes underlying human complex traits.

**Supplementary Information:**

The online version contains supplementary material available at (10.1186/s13059-021-02347-6).

## Introduction

The complexity of the human genome lies not only in its composition of billions of base pairs, but also in the chemical modifications that make it interpretable to enzymes and other molecular factors, through epigenetic mechanisms. DNA methylation has been the most widely studied epigenetic mark since 1948 when it was first reported [1]. In humans, DNA methylation consists of the covalent addition of a methyl group to cytosine residues—predominantly at CpG sites—by a family of enzymes called DNA methyltransferases (DNMTs) [2, 3]. DNA methylation plays an important role in multiple processes during human development and over the life course, such as the regulation of transcription [4–6], genomic imprinting [2, 4], maintenance of X-chromosome inactivation [7], chromosomal maintenance, and genomic stability [8].

With advances in high-throughput molecular techniques our understanding of DNA methylation has greatly increased in the past few decades. Multiple methods have been developed for profiling DNA methylation patterns across the human genome. Currently, the gold standard is bisulfite conversion of DNA followed by deep sequencing or whole genome bisulfite sequencing (WGBS, Table 1). However, the most extensively used methylation profiling technologies are microarrays assessing DNA methylation at a proportion of the 28 million CpG sites in the genome. To date, Illumina bead-chip platforms have been most popular, where pre-designed probes target bisulfite-converted DNA, followed by hybridization, single-base extension, and its detection [9]. Early models included arrays such as the Infinium HumanMethylation27 BeadChip (27K), targeting around 27,000 sites (0.1% of total CpGs) mainly in CpG islands (CGIs) within promoters [9], followed by the widely used Infinium HumanMethylation450 array (450K), targeting ∼480,000 sites (1.7% of total CpGs) consisting of the 27K sites and increased coverage in non-CGIs and intergenic regions [10]. A more recent version is the Infinium MethylationEPIC BeadChip (EPIC), targeting ∼850,000 sites (3% of total CpGs), which include almost all of the 450K sites, with additional CpG sites in enhancers [11].

**Table 1:** Glossary of commonly used terms

Term	Definition
**Additive genetic effects**	A genetic mechanism where alleles at one or more loci have a cumulative contribution to the phenotype. In human **QTL** analysis. An additive genetic model describes a model where the mean phenotype value changes by *n* units in heterozygotes and by 2*n* in homozygotes, with each additional copy of the risk allele at a locus. In heritability models, the contribution of additive genetic effects to the phenotype variance is estimated by the **narrow-sense heritability**.
**Allele-specific methylation (ASM)**	Allelic asymmetry in DNA methylation status at a locus. ASM can be a consequence of several factors, such as genetic variation (**sequence-dependent ASM**, a type of **meQTL** effect) or genomic imprinting.
**CpG island (CGI)**	Region of the genome where the frequency of CpG sites is greater than that expected by chance. Different definitions of CGI have been proposed. CGIs are flanked by regions known as **CGI shores** and **shelves**.
**Differential methylation analysis**	Computational analysis that aims to identify a statistically significant difference in mean DNA methylation levels across sample groups. The approach can be applied to individual CpG sites (differentially methylated sites or positions, **DMSs** or **DMPs**) or to multiple consecutive CpG sites (differentially methylated region, **DMR**).
**DNA methylation microarray**	Microarray-based technology that quantifies DNA methylation levels at a pre-specified set of CpG sites. Commonly used approaches typically apply bisulfite conversion of the DNA, followed by Illumina DNA methylation array profiling. Illumina methylation arrays include the Infinium HumanMethylation27 (**27K**), HumanMethylation450 (**450K**) and MethylationEPIC (**EPIC**) BeadChips. Different arrays profile different proportions of the **methylome** (**coverage**).
**Epigenome-wide association study (EWAS)**	Analysis that systematically assesses the association between epigenetic marks (e.g., DNA methylation levels) at genetic loci across the genome and a phenotype or exposure of interest.
**Genome-wide association study (GWAS)**	Analysis that systematically assesses the association between genetic variation at genetic loci across the genome and a phenotype of interest.
**Heritability**	The proportion of variance in a phenotype that is attributed to the genetic variation. The **broad-sense heritability** describes the subset of phenotype variance due to all genetic effects, while the **narrow-sense heritability** describes the proportion of phenotype variance only due to **additive genetic effects**.
**Methylation quantitative trait locus (meQTL)**	A genetic locus at which genetic variation is associated with variation in DNA methylation at a specific CpG site. MeQTLs can form local associations in ***cis***, or have long-range effects in ***trans***.
**Methylome**	The DNA methylation profile of the genome. The methylome can be profiled at different levels of resolution, in single cells or in populations of cells, across different cells and tissues, and at a specific moment in time. It can be profiled using different technologies including **WGBS** and **DNA methylation microarrays**.
**Multiple-testing correction**	When multiple simultaneous statistical tests are carried out, the probability of spurious discoveries increases. Different multiple testing correction procedures can be applied, including methods that control the false discovery rate (**FDR**) and the family-wise error rate (**FWER**), for example, Bonferroni correction.
**Pleiotropy**	A genetic variant that impacts multiple phenotypes.
**Post-GWAS analysis**	Follow-up analysis from **GWAS** that aims to characterize the functional role of GWAS signals and/or identify causal genetic variants. Many QTLs have been identified for variation in molecular processes, including but not limited to DNA methylation (**meQTL**), gene expression (**eQTL**) and histone modifications (**hQTL**).
**QTL effect**	A quantitative measure that describes the strength and direction of association between a genetic variant and its associated phenotype, estimated in genetic association analysis.
**Whole genome bisulfite sequencing (WGBS)**	Deep sequencing technology used to detect the methylation status of all sites in the **methylome**. WGBS consists of bisulfite conversion of the DNA, followed by whole genome sequencing.

Unlike DNA sequence, genomic methylation patterns are not directly inherited during meiosis [12], but are mostly reprogrammed in two waves during embryogenesis [13–15]. Following this, DNA methylation modifications can be both stable and dynamic during mitosis events that accumulate over the life course [16, 17]. These observations suggest that the environment may be a key driving force behind changes in mitotic DNA methylation [17–20]. However, growing evidence now shows that genetic variation also plays a role in the establishment of DNA methylation marks, independently of or in contribution with environmental exposures.

Research interest in genetic impacts on DNA methylation variation is especially relevant in context of methylome changes observed in disease [16, 21–23], alongside results from genome-wide association studies (GWASs). Although many genetic associations have been identified from GWASs, there remain important unanswered questions about candidate causal variants and their functional consequences, as GWAS signals tend to fall in non-coding regions [24]. Methylome analyses can provide a valuable piece of information as a post-GWAS resource, giving insights into regulatory genomic potential of GWAS signals and helping to prioritize loci to further follow-up [25–27].

Given these considerations, here, we present an overview of results identifying genetic drivers of DNA methylation variation. We discuss methylation heritability findings, and then focus on genome-wide studies that have identified genetic variants as meQTLs for DNA methylation profiles. We also discuss cellular mechanisms that may explain genetic impacts on DNA methylation levels. Lastly, we consider challenges of meQTL analyses, as well as novel applications and further research directions.

## DNA methylation heritability

A fundamental question in the study of human traits is to assess the extent to which a phenotype is under the influence of genetic factors, that is, how *heritable* it is. Heritability refers to the proportion of phenotypic variance attributed to either total genetic effects (broad-sense heritability, *H*^2^), or additive genetic effects (narrow-sense heritability, *h*^2^) [28], where the latter is most commonly estimated in context of DNA methylation analyses.

For the estimation of DNA methylation heritability, most studies apply twin-based study designs. The underlying premise of the twin design is based on trait comparison between monozygotic (MZ) twin pairs who share typically 100% of their genome variation, compared to dizygotic (DZ) twins who share on average only 50% of genetic variation. The narrow-sense heritability is then calculated by comparing the correlation of a trait—here level of DNA methylation at a genomic region—between MZ and DZ twins [29], following a series of assumptions. In a recent study in whole blood samples from 2603 individuals from the Netherlands Twin Registry, van Dongen et al. [30] estimated the individual CpG site heritability to range from 0 to 0.99 at each CpG site profiled on the Illumina 450K array, where the mean genome-wide heritability averaged over all CpG sites tested was $\hat {\bar {h^{2}}}=0.19$ ($\hat {\bar {h^{2}}}=0.20$ with the classical twin method). The estimate of the average CpG site heritability across the methylome as 0.19 is in agreement with previous twin methylation heritability studies using the 450K [31] and 27K arrays [32]. Furthermore, the study estimated that approximately 41% of Illumina 450K sites had significant evidence for additive genetic effects and suggested that heritability at a proportion of DNA methylation sites is sex- and age-specific.

Fewer studies estimate DNA methylation heritability using other approaches, for example, using familial clustering models in extended families. The advantage of such methods is their wider applicability to multiple types of relatives beyond twins and circumventing key assumptions of the classical twin model such as equal influence of common environment for MZ and DZ twins and independence of genetic and environmental factors. Despite this, DNA methylation heritability estimates from familial clustering studies are consistent with those obtained from classical twin models. McRae et al. [33] estimated the heritability of DNA methylation measured using the Illumina 450K array in 614 peripheral blood leukocyte samples from twin pairs, their siblings and fathers, in altogether 117 families of European descent from the Brisbane System Genetics Study. The estimates of heritability across the Illumina 450K probes give a similar mean CpG site genome-wide estimate of $\hat {\bar {h^{2}}}=0.187$, ignoring probes with known genetic variants ($\hat {\bar {h^{2}}}=0.199$, if all probes included). Using a different approach, Nustad et al. [34] designed a Bayesian mixed model that could include pedigree structure for estimating heritability in two sets of CD4^+^ T cell samples (*n*=995 and *n*=530) from the Genetics of Lipid Lowering Drugs and Diet Network (GOLDN) study, profiled on the 450K array. Here, the mean heritability point estimates across the genome ($\hat {\bar {h^{2}}}=0.33$ and $\hat {\bar {h^{2}}}=0.36$) are slightly higher compared to other studies, potentially because the mean was calculated only considering CpG sites with strong evidence for non-zero heritability, as well as lack of precise estimates of shared environmental effects. Other studies using the 450K array have found comparable average heritability estimates based on family clustering ($\hat {\bar {h^{2}}}=0.09$ [35] and $\hat {\bar {H^{2}}}=0.13$ [36]), or other methods applicable to unrelated individuals such as SNP-based heritability, calculated using all genetic variants [30, 37]. For instance, in 3948 blood samples from the Avon Longitudinal Study of Parents and Children (ALSPAC), Gaunt et al. [37] estimated the genome-wide average SNP-based heritability for 450K array probes in blood at different time points over the life course to range between 0.20 and 0.25, based on a panel of 1.2 million common SNPs. The majority of methylation variance was explained by SNPs located over 1 Mbp away from the methylation site (or in *trans*).

Overall, these heritability studies indicate that DNA methylation profiles have a genetic basis, which expressed as the average heritability across all CpG site in the genome profiled by the Illumina 450K array, ranges from 0.1 to over 0.3. Although this genome-wide mean estimate of methylation heritability could be considered moderate or low, the heritability distribution at specific CpG sites ranges from 0 to 1, and at least one tenth of profiled sites are highly heritable ($\hat {h^{2}} > 0.5$) [30, 31, 33]. Furthermore, because genetic variability differs across populations and over time, heritability estimates are population- and age-specific, which may explain some of the differences in reported mean DNA methylation heritability estimates so far [28]. Another factor to consider when interpreting the heritability estimates is that they may vary according to DNA methylation platform [38, 39], as array technologies only cover a limited proportion of CpGs out of the 28 million CpGs genome-wide (approximately 1.7% for 450K, 3% for EPIC) and regulatory elements tend to be underrepresented (see the “DNA methylation profiling” section).

An outstanding research question has considered evidence for transgenerational transmission of DNA methylation patterns independent of genetic variation or transgenerational epigenetic inheritance. In model organisms such as mice and rats, several phenotypes have been linked to DNA methylation transgenerational inheritance. Examples include a kinked tail phenotype caused by methylation in a retrotransposon within the axin-fused allele in mice [40], and metabolic phenotypes in male rats linked to in utero nutritional deficiencies and alterations in the sperm methylome [41]. In contrast, human transgenerational epigenetic inheritance studies are limited and show negative results, suggesting that genetic variants likely fully explain the observed methylation heritability. In a study aiming to test whether methylation levels at certain CpG sites are inherited in a Mendelian fashion through multiple generations in 16 families (123 subjects) from the Arab population, Zaghlool et al. [42] inspected loci where blood DNA methylation levels followed a trimodal distribution, that is, with peaks around 0 (unmethylated), 0.5 (hemi-methylated), or 1 (methylated). Although about a thousand CpG sites from the 450K followed such patterns, in almost all cases, DNA methylation changes were associated with nearby genetic variants (within 1 Mbp or less), discarding a direct mechanism of transmission that is independent of genetic variation. Importantly, the trimodal loci had high mean heritable values (0.8±0.18), and almost half were associated with expression quantitative trait loci (eQTLs). McRae et al. [33] reached similar conclusions, noting that the transgenerational inheritance of DNA methylation is mainly attributable to genetic heritability. Therefore, so far, there is no robust evidence in humans to indicate that DNA methylation heritability may be attributed to non-genetic effects, such as evidence for transgenerational epigenetic inheritance as reported in other species [12].

## Methylation quantitative trait loci

Given the observed evidence for DNA methylation heritability, much interest has focused on identifying specific genetic variants that influence DNA methylation variation across the genome. Multiple studies have explored the correlation between DNA methylation levels and genetic variants across the genome (typically single nucleotide polymorphisms, SNPs), to identify DNA methylation quantitative trait loci or meQTLs (also referred to as mQTLs or metQTLs). Although several early papers tackled meQTLs identification over limited target sites [43–46], it was not until the early 2010’s that initial genome-wide efforts identified meQTLs on the 27K methylome and across multiple tissues (Gibbs et al. [47], Zhang et al. [48] and Bell et al. [49]).

Studies to date have reported an influence of meQTLs on up to 45% of CpG sites profiled by the Illumina 450K array across the genome [31, 35, 50, 51], with more than 90% of meQTLs acting on nearby methylation sites (in *cis*) [38, 50, 52]. CpG sites that have higher heritability estimates are more likely to be associated with meQTLs in *cis*, *trans*, or both, and have a clear polygenic architecture [35, 38, 50]. Some studies also include replication in independent sample sets, although overall a direct comparison of meQTL signals can be challenging because studies do not systematically report meQTL effect sizes. Despite observations that meQTLs tend to have moderate to large effects, the “missing heritability” issue has also been raised in the context of meQTLs. That is, family-based heritability estimates of DNA methylation are greater than the proportion of variance explained by meQTLs, especially for distal associations [37, 53–56].

### Detecting meQTLs

**Fig. 1:**
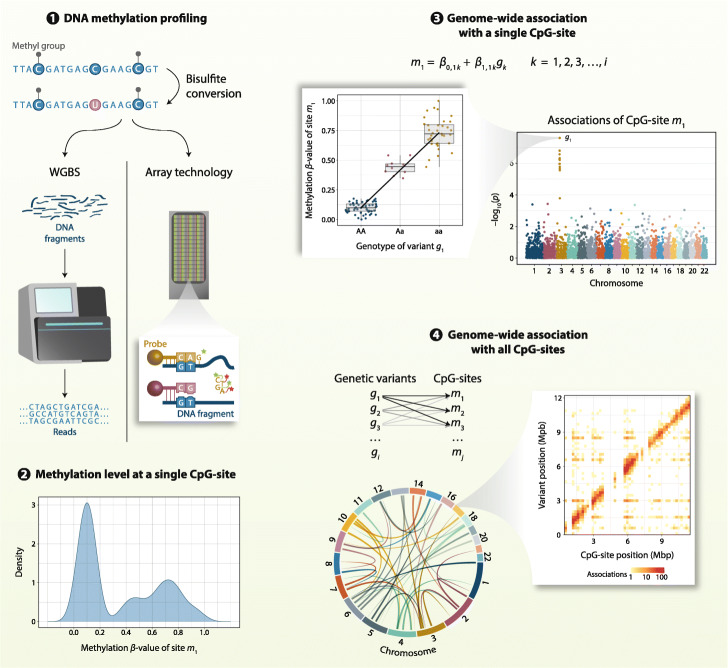
A typical workflow for meQTL identification. Step **1** is DNA methylation profiling. The most commonly applied methylation profiling technologies in meQTL studies are Illumina methylation arrays and whole genome bisulfite sequencing (WGBS). In both approaches, DNA is treated with bisulfite, converting unmethylated cytosines into uracils, and leaving methylated cytosines unchanged. DNA can then be profiled by sequencing or by Illumina array technologies, consisting of pre-designed probes. In step **2**, DNA methylation levels at each CpG site are quantified, typically either as percentage (0–100%, e.g., in WGBS) or proportion methylation (0–1, e.g., in the Illumina technology methylation *β*-value). The example shows the distribution of methylation *β*-values for one CpG site (*m*_1_) across all profiled samples. Step **3** is the association of a set of genetic variants (coded as allele dosages at each locus) with methylation values at each CpG site, usually using linear models. In this example, after the association test at site *m*_1_ with a set of *i* genetic variants (shown in the Manhattan plot), *g*_1_ was found to be significantly associated with *m*_1_ (shown in the boxplot). Finally, step **4** represents the extension of the genetic association test to all profiled CpG sites genome-wide and the identification of genome-wide meQTLs after setting an appropriate threshold for statistical significance. The resulting meQTL associations can be either short-range, in *cis* (shown in heatmap for a few Mbp), or long-range or on different chromosomes, in *trans* (shown in Circos plot with all chromosomes)

MeQTL identification is based on association tests between genetic variation genome-wide and DNA methylation levels at a specific CpG site (Fig. 1). As for other quantitative trait analyses, the majority of meQTL detection approaches apply linear models, where the DNA methylation level at a CpG site is the response variable and genetic variants are predictors along with technical and biological covariates, such as smoking and age. Other statistical tests employed include non-parametric methods such as Spearman rank correlation [51, 57–59] and Kruskal-Wallis rank test [60], that do not make assumptions about the distribution of variables, or even machine learning approaches such as random forests [61].

In most studies the focus is on detecting evidence for additive genetic effects alone, where the genetic predictor is the dose of the alternative allele, for example, 0 for genotype “AA”, 1 for “Aa” and 2 for genotype “aa”. To date and to our knowledge, full genome-wide meQTL analyses have not yet considered genetic association models including dominance effects or overall genotype effects. However, Zeng et al. [62] explored meQTLs at 984 CpGs with parent-of-origin effects (POE) in 5101 individuals from Scottish families. The model included additive effects (coded as the dosage of alternative allele), dominance effects (coded 1 for heterozygotes and 0 for homozygotes), and POE effects (coded 0, −1, and 1 for homozygotes, “Aa” and “aA”, respectively). Likewise, some studies focusing on subsets of CpGs have identified meQTLs in gene interaction models, specifically gene-by-gene (G ×G) and gene-by-environment (G ×E) (see the “Gene–environment interactions” section).

The majority of studies discussed here apply Illumina DNA methylation arrays. In these platforms, the DNA methylation level at a CpG site is quantified through the Illumina methylation *β*-value, defined as the intensity measured in the methylated probes for that CpG over the total intensity across all probes for the CpG and a constant. The methylation *β*-value is often interpreted as the probability of methylation at a given site, or the proportion of methylated cells in the sample (Fig. 1). Some studies apply transformations of methylation *β*-values—such as the logit transformation or *M*-value—which are more appropriate to control for heteroskedasticity but are perhaps less biologically interpretable [63, 64].

In addition to meQTL studies that explore DNA methylation levels using Illumina arrays, several meQTL approaches have also applied sequencing technologies. To date, only one study has used sequencing techniques to detect meQTLs across the full genome, rather than focusing on specific genomic subsets. In a sample of 697 Swedish subjects, McClay et al. [52] used methyl-CpG-binding domain (MBD)-enriched sequencing (MBS-seq) genome-wide and profiled ∼13M CpGs collapsed into 4.5M loci across the genome. DNA methylation was quantified by estimating the coverage at each CpG. The results show that 15% of methylation loci have meQTLs (primarily within 1 Mbp), and 98% of the tested SNPs were associated with at least one CpG. Other studies have employed strategies such as targeted bisulfite sequencing of a pre-designed panel with informative genomic regions [65], MeDIP-sequencing at candidate regions [66], and meQTL replication in WGBS data [67]. Several studies have also explored sequence-dependent allele-specific methylation (ASM), which represents a specific type of meQTL effect in *cis*. In contrast to meQTL analysis, ASM discovery is restricted to heterozygous regions within single samples, and comparison of differentially methylated CpG sites (DMSs) between the two distinct alleles, for example, using Fisher’s exact test or equivalent. ASM studies to date have been carried out using bisulfite sequencing in a moderate number of samples (less than 100). ASM results show that around 10% of the explored CpGs exhibit allelic imbalance at heterozygous regions [65, 68–70], which is consistent with meQTLs results.

### Distribution of meQTLs across the genome

#### MeQTLs can have local or distal effects

MeQTLs can be divided into two classes based on the proximity of the genetic variant to the CpG site. *Cis*-meQTLs are genetic variants near to or proximal to the target CpG site, and *trans*-meQTLs are separated by one or more Mbp from the target CpG or located on different chromosomes. Identification of *cis* and *trans*-meQTLs includes testing for associations across all possible pairs of SNPs-CpGs. Pairs can be categorized into “proximal” or “distal” and multiple testing correction can be applied for each group independently, or they can be analyzed together and annotated *post hoc* [38, 47, 71]. Correcting for multiple testing burden is a crucial step for the definition of genome-wide significant *p*-value thresholds. Published thresholds are typically of the order of *p*<1×10^−5^ for *cis* effects and *p*<1×10^−9^ for *trans* effects, based on applying permutation-based approaches to estimate the false discovery rate (FDR) or Bonferroni correction to control the family-wise error rate (FWER). The exact multiple testing correction threshold clearly depends on the methylation array and genotype coverage, methylation, and genotype distributions, as well as sample structure and sample size if permutation-based approaches are applied (see the “Multiple-testing correction” section). Some studies limit the search to *cis*-meQTLs alone, reducing the number of total tests, or carry out *trans* associations only for selected SNP-CpG pairs [51, 60].

To date, the primary focus has been on *cis*-meQTLs identification. In general, studies with large sample sizes (> 1000) have estimated that at least 10% [38, 53–55] and up to 45% [35, 50, 51] of the methylome is influenced by nearby meQTLs. A consideration in *cis*-meQTL analysis protocols is the maximum distance between genetic variants and CpG sites. Published studies have applied a range from a few kbp to 1 Mbp, but in almost all cases it has been observed that the strength of the *cis*-meQTL effect is inversely proportional to the distance between genetic variant and CpG site. For example, in one of the early genome-wide meQTL analysis using 27K DNA methylation levels in lymphoblastoid cell lines (LCLs), 37 CpG sites had meQTLs in genome-wide analyses across all possible SNP-CpG pairs, but for 27 of these sites the most significant meQTL was located within 50 kbp of the CpG site [49]. More recently, Hannon et al. [38] conducted a genome-wide analysis across all SNPs-CpGs using EPIC DNA methylation levels in 1111 blood samples. The results identified meQTLs at 12% of assayed methylation sites, and again a predominance of these associations occurred in *cis*. Higher effect sizes were observed for genetic variants within a maximum of 500 kbp from the CpG site (in *cis*), where the average of the change in DNA methylation per allele was of 3.48%, compared to 3.26% in *trans*.

Conversely, meQTL genome-wide association analyses to date agree that no more than 5% of total CpGs show evidence for *trans*-meQTLs. The exception to this are the results from Gong et al. [72] estimating meQTLs in different cancer tissues samples. The observation of higher *trans*-meQTL proportions here (more than 10% of total CpG sites are associated with *trans*-meQTLs in eight cancer types) suggests that under certain conditions, the effects of the distal associations could be enhanced. Furthermore, although *trans*-meQTL are relatively rare genome-wide, these effects also tend to target specific genomic regions (see the “MeQTLs are differentially distributed across the genome” section).

The physical threshold for categorizing a meQTL as *cis* or *trans* matters. Insights into the distance between *cis*-meQTLs and the target CpG sites were gained by Banovich et al. [67] who used a relatively small *cis* window of 6 kbp to detect meQTLs in LCLs. The authors estimated the median distance of putative causal *cis* associations as 76 bp, with 87% of the meQTLs located within 3 kbp of the CpGs. At the other end of this spectrum, Huan et al. [35] report that 70% of intra-chromosomal *trans*-meQTLs were within 5 Mbp of the target CpG, leading to the conclusion that such associations may act as long-range *cis*-meQTLs, rather than as *trans*. In contrast, inter-chromosomal associations are the most commonly reported *trans*-meQTLs, accounting for at least 65% of the *trans*-meQTLs [35, 37, 50, 51, 55]. Another factor to take into account is that some *trans* associations could be SNPs in long-range linkage disequilibrium (LD) with “real” *cis*-meQTLs—as observed for 17% of intra-chromosomal SNP-CpG associations in lung tissue, after conditional analysis [56].

#### MeQTLs are differentially distributed across the genome

Early efforts exploring the correlation between genetic variants and DNA methylation showed evidence that meQTLs and their target DNA methylation sites are not randomly distributed in the genome. Non-genic regions and enhancers appear to be hotspots for CpG sites associated with *cis*-meQTLs, while CpG islands (CGIs), 5 ^′^ untranslated regions (UTRs) and regions upstream of the transcription start sites (TSSs) show depletion of CpG sites with *cis*-meQTLs. In contrast, the opposite pattern is observed for CpGs with *trans*-meQTLs, which are enriched in CGIs and in promoters and regions surrounding the TSSs, and are underrepresented in gene bodies, 3 ^′^UTRs, and heterochromatin regions [35, 37, 50, 51, 53, 56, 67, 73]. This genomic distribution of meQTL-related CpGs appears to be quite stable during several life stages [37] and across tissues [73].

The underrepresentation of CpGs with *cis*-meQTLs in CGIs is related to the observation that most of the tested CpGs in CGIs fall in gene promoters, where they tend to be constitutively hypomethylated and have lower DNA methylation variances [5, 6]. As hypothesized by Do et al. [70], meQTL-associated CpGs may be located in areas with more flexible evolutionary constrains, in contrast to typically hypomethylated CGIs which are conserved across vertebrate promoters [74]. This hypothesis is also supported by results from Husquin et al. [60] who observed that DMSs in monocytes between two populations (78 samples of African descent and 78 of European descent) are enriched to harbor *cis*-meQTLs (70.2% of DMSs have *cis*-meQTLs) compared to the genome-wide meQTL proportion (12.6% of EPIC sites had *cis*-meQTLs). Hence, CpGs, where methylation patterns are less conserved across different populations, have a higher probability of being under the influence of meQTLs.

The genetic variants driving meQTL effects also exhibit non-random genomic distributions. Min et al. [50] found that active chromatin domains and genic regions were enriched for meQTLs that act in *cis* only or both in *cis* and *trans*, while heterochromatin and intergenic regions were enriched for *trans* only meQTLs. Using a different approach, an analysis at 11.5 million DNA methylation sites profiled by WGBS in 34 samples [75] identified 221 de novo DNA motifs associated with unmethylated regions, and 92 motifs associated with methylated regions. Using data from previously published studies, the authors found that DNA motifs associated with methylation were enriched in meQTLs variants, especially near TSSs.

Lastly, *trans*-meQTLs results show that the number of inter-chromosomal *trans*-meQTLs is usually proportional to the number of genes in a chromosome, except for chromosomes 16 and 19 which are highly enriched for *trans*-meQTLs and chromosome 1 which is depleted for *trans*-meQTLs [37, 53]. Also, McRae et al. [53] estimated that almost 25% of *trans*-meQTLs are located in telomeres and sub-telomeres. The major histocompatibility complex (MHC) region is another locus that harbors highly heritable CpGs and meQTLs associated with multiple CpGs [33, 76, 77].

### Tissue-specificity of meQTL effects

DNA methylation plays an important role in cell lineage and tissue differentiation, resulting in tissue-specific methylation profiles over a considerable proportion of the methylome. Most meQTL studies explore whole blood, but analyses within specific cell types or bulk tissue have also been carried out.

#### MeQTLs in blood-based samples

Most studies have identified meQTLs in blood and blood-derived cells, including whole blood, LCLs, peripheral blood mononuclear cells (PBMC), and leukocytes (see Table 2). Blood-based meQTLs studies are most common to date, have larger sample sizes, and have shown high replicability. The majority of blood reports are not limited to the discovery of novel meQTLs alone, but also include study designs that integrate DNA methylation findings with GWAS results or other biological data. In the largest study to date, the Genetics of DNA Methylation Consortium (GoDMC), a multi-cohort meta-analysis meQTL resource, combined data from 32,851 blood samples across different population cohorts and found that 45.2% of CpGs in the 450K array have meQTLs, with greater effect sizes for *cis* associations [50]. Additionally, the authors detected substantial sharing between meQTLs and GWAS signals, and constructed a network of CpG sites that share meQTLs, identifying 405 highly interconnected genomic communities enriched for regulatory genomic features and links to complex traits. Huan et al. [35] performed an analysis in 4170 whole blood samples, identifying 4.7 million *cis*-meQTLs (within 1 Mbp of target CpG) and 706 thousand *trans*-meQTLs. After a follow-up analysis, the authors found 92 CpGs with a likely causal role in cardiovascular disease, as well as supporting evidence of CpG-expression contribution to these putative causal pathways. Likewise, Bonder et al. [51] studied *trans*-meQTLs focusing on SNPs previously associated with complex traits. Using 3841 whole blood samples from the Netherlands, they showed that one-third of the analyzed SNPs affect DNA methylation levels at 10,141 CpG sites in *trans*, and where 95% of *trans*-meQTLs were validated in external data from 1748 lymphocytes. Furthermore, the authors provided several examples of *trans*-meQTLs with effects on specific transcription factors levels as well as methylation of their binding sites across the genome. Chen et al. [78] identified *cis*-meQTLs in immune cells (CD14^+^ monocytes, CD16^+^ neutrophils, and naive CD4^+^ T cells) at almost 10% of the CpG sites from the Illumina 450K, and estimated relatively low blood cell specificity of meQTLs especially between myeloid cells.

**Table 2:** Blood-based genome-wide meQTL studies (sample size > 100) in whole blood or blood-derived cell samples

Ref.	Sample size^a^	Methylation assay	Genotyping assay	Significant CpGs^b^	Significance threshold^c^	Remarks
[50]	27,750	Illumina 450K	1000G^d^	*cis* (≤ 1 Mbp), 43.5% *trans* (> 1 Mbp), 4.5%	*p*_*cis*_<1×10^−8^,*p*_*trans*_<1×10^−14^	Study design: two-phase meta-analysis in a total of 36 cohorts. Additional analysis: replication of 188,017 meQTLs in external sample (76% for *cis* and 79% for *trans*).
[35]	4170	Illumina 450K	Affymetrix 500K and 50K MIP, 1000G^d^	*cis* (≤ 1 Mbp), 29.3% *trans* (> 1 Mbp), 3.3%	FWER^1^ < 5% (*p*_*cis*_<2×10^−11^,*p*_*trans*_<1.5×10^−14^)	Additional analysis: replication of effect direction in two external samples (81–99%).
[136]	429	Illumina 450K	GOLDN study^e^	*cis* (≤ 1 Mbp), 0.3%	FWER^1^ < 5% (*p*<5.6×10^−10^)	Study design: response meQTL, with log transformed post-/pre-treatment methylation values.
[38]	1111	Illumina EPIC	Illumina CoreExome, 1000G^d^	*trans*, 12.2%	FWER^1^ < 5% (*p*<6.5×10^−14^)	Effect size: mean methylation change per additional reference allele of 3.46% (*s*=3.01*%*).
[60]	156 [monocytes] (two samples)	Illumina EPIC	Illumina Omni, WGS, 1000G^d^	*cis* (≤ 100 kbp), 12.6%	FDR^3^ < 5% (*p*<1×10^−5^)	Study design: separate meQTLs discovery for the two samples, joint results reported. Additional analysis: *trans* association restricted to SNPs in TFs genes vicinity.
[53]	1980 (two samples)	Illumina 450K	Illumina 610-Quad	*cis* (≤ 2 Mbp), 15.4–15.7% *trans* (> 2 Mbp), 0.5–0.6%	FWER^1^ < 5% (*p*_*cis*_<1×10^−11^,*p*_*trans*_<1×10^−13^)	Study design: separate meQTLs discovery for the two samples. Additional analysis: replication of CpGs in both samples (13.3% for *cis* and 0.5% for *trans*).
[93]	337	Illumina 450K	Illumina CytoSNP, 1000G^d^	*cis* (≤ 500 kbp), 18.3%	FDR^3^ < 1%	
[157]	729	Illumina 450K	Illumina Exome, Hap300 and Omni, 1000G^d^	*cis* (≤ 500 kbp), 12.1%	FWER^1^ < 5% (*p*<9.4×10^−11^)	Additional analysis: variance meQTLs.
[85]	460 [cord blood and whole blood] (two samples)	Illumina 450K	Illumina Omni, 1000G^d^	*cis* (≤ 0.5–1 Mbp), 8.1–19.2%	FDR^3^ < 5%	Study design: separate meQTLs discovery for two samples with different parameters. Additional analysis: meQTLs co-localization with results from two published studies.
[51]	3,841	Illumina 450K	CODAM34, LLD9, LLS38, NTR12, and RS studies^e^, GoNL^d^	*cis* (≤ 250 kbp), 34.4%	FDR^3^ < 5% (*p*<1.4×10^−4^)	Additional analysis: *trans* association restricted to 6111 informative SNPs.
[65]	744 [T cells and whole blood] (two samples)	MCC-seq, WGBS	WGS, Illumina Omni, inferred from WGBS, 1000G^d^	*cis* (≤ 250 kbp), 16.0–18.1%	FDR^4^ < 10%	Study design: separate meQTLs discovery for the two samples. Additional analysis: meQTL in visceral adipose tissue samples, ASM analyses and genotype-independent tests, and validation on Illumina 450K.
[78]	525 [neutrophils, monocytes and T cells] (three samples)	Illumina 450K	WGS	*cis* (≤ 500 kbp), 9.9%	FWER^1^ < 5%	Study design: separate meQTLs discovery for the three samples, mean results reported.
[37]	3948 [cord blood and whole blood] (five samples)	Illumina 450K	Illumina Hap550 and 660W, 1000G^d^	*cis* (≤ 1 Mbp), 6.1–8.0% *trans*(>1 Mbp), 0.5–0.7%	FWER^1^ < 0.2% (*p*<1×10^−14^)	Study design: separate meQTLs discovery for the five samples. Additional analysis: replication of CpGs in pairwise comparisons (83–98% for *cis* and 88–98% for *trans*).
[54]	850	Illumina450K	Illumina Hap550, Exon510, 1M and 1M-Duo	*cis* (≤ 50 kbp), 13.8%	FWER^1^ < 5% (*p*<2.6×10^−9^)	
[55]	1748 [lymphocytes] (cancer-case and control samples)	Illumina 450K	OFCCR^e^, Affymetrix 500K	*cis* (≤ 1 Mbp), 13.9% *trans*(> 1 Mbp), 0.5%	FDR^4^ < 5% (*p*_*cis*_<4.8×10^−10^,*p*_*trans*_<3.2×10^−13^)	Study design: joint meQTLs discovery for the two samples; *trans* analysis excluded CpG sites with *cis* meQTLs. Additional analysis: replication of meQTL-CpG pairs in two external samples (83.6% for *trans*).
[52]	697	MBD-seq	Affymetrix SNP 5.0 and 6.0, Illumina Omni, 1000G^d^	*cis* (≤ 1 Mbp), 15% *trans*(> 1 Mbp), 0.1%	FDR^4^ < 1%	Additional analysis: replication of findings in one sample of schizophrenia cases (*π*1= 95% for *cis*, 98.7% for *trans* same chromosome, and 99.3% for *trans* different chromosome).
[86]	264 [cord blood and whole blood] (three samples)	Illumina 27K	Illumina Omni, Affymetrix SNP 5.0 and 6.0, HapMap^d^	*cis* (≤50 kbp), 0.7–1.5%	FWER^2^ <5%	Study design: separate meQTLs discovery for the three samples. Additional analysis: replication of meQTL-CpG pairs in pairwise comparisons (17.8–69.5%); meQTL in four brain regions samples.
[59]	177 [T cells and LCL]	Illumina 450K	Illumina Omni, 1000G^d^	*cis* (≤ 5 kbp), 5.4–7.8%	FDR^3^ < 10%	Study design: separate meQTLs discovery for the two samples. Additional analysis: meQTL in fibroblasts sample.
[32]	171	Illumina 27K	Illumina Hap300, 610-Quad, 1M-Duo and 1.2M-Duo, HapMap^d^	*cis* (≤ 50), 6.3%	FDR^3^ < 5% (*p*<1×10^−5^)	

^a^If not specified, the sample type is whole blood. If more than one sample per analysis, the pooled size and number of samples is reported

^b^In parenthesis, maximum or minimum distances are indicated for *cis* and *trans* analysis, respectively. The range of results is presented if more than one analysis was done (unless otherwise stated)

^c^Multiple-testing criteria, with the corresponding *p*-value threshold for *cis* and *trans* meQTLs (where it differs). Different approaches to estimate FWER and FDR are as follows:

^1^FWER based on Bonferroni correction

^2^FWER based on Holm-Bonferroni correction

^3^FDR based on permutations

^4^FDR based on Benjamini-Hochberg correction

^d^Reference panel for imputations

^e^Database or biobank

*FWER* family-wise error rate, *FDR* false discovery rate, *LCL* lymphoblastoid cell lines, *WGS* whole genome sequencing, *MCC-seq* methylC-capture sequencing, *WGBS* whole genome bisulfite sequencing, *MBD-seq* methyl-CpG-binding domain sequencing, *1000G* 1000 genotypes, *GoNL* Genome of the Netherlands, *TF* transcription factor, *ASM* allele-specific methylation

#### MeQTLs in non-blood-based tissues and cells

Genome-wide meQTLs have also been identified in a range of tissues including several regions of the brain, lung, skeletal muscle, buccal and saliva samples, placenta, and adipose tissue (Table 3). The discovery of meQTLs across brain regions [57, 71, 79, 80], their overlap with non-brain tissue findings [70, 73] (see the “Tissue-shared meQTLs-CpGs” section), and their co-localization with other molecular QTLs [81] has initiated further studies to identify and characterize the role of genetic variants underlying neurological disorders. In lung tissue, Morrow et al. [82] investigated meQTLs that may impact the pathogenesis of chronic obstructive pulmonary disease in 90 cases and 36 controls. The authors found *cis*-meQTLs at 10% of the 450K CpGs, and significant overlaps with GWAS signals for the disease. In parallel, Taylor et al. [83] assessed 282 samples of skeletal muscle on the Illumina EPIC array and found *cis*-meQTLs for almost 21% of CpGs. In adipose tissue, Grundberg et al. [31] (*n*=603, from UK females) and Volkov et al. [76] (*n*=119, from Scandinavian males) identified the *cis* and *trans* genetic effects on the methylome profiled by the 450K array. Both studies identified meQTLs that may also be involved in metabolic traits, such as variants in the *ADCY3* gene, associated with obesity and BMI.

**Table 3:** Overview of published genome-wide DNA methylation quantitative trait loci studies in blood-independent sample types

Sample type	Association	Methylation	Sample sizes^b^	Significant	Significant	Ref.
	analyses^a^	assays		*cis*-CpGs^b^	*trans*-CpGs^b^	
Brain	18	Illumina 450K,	18–468	0.1–13.6%	0.1–5.1%	[47, 48, 57, 70, 71, 79, 80, 86, 174]
		Illumina 27K				
Buccal	2	Illumina EPIC	86–197	4.3–7.4%		[39, 73]
Cancer	23	Illumina 450K	103–664	1.7–6.4%	0.1–24.8%	[72]
Connective tissue (adipose, fibroblasts)	4	Illumina 450K,	107–603	3.2–28.5%	0.1%	[31, 59, 65, 76]
		MCC-seq				
Epithelial	1	Illumina 450K	111	4.4%		[175]
Lung	2	Illumina 450K	126–210	10–10.1%	0.2%	[56, 82]
Placenta	2	Illumina 450K	37–303	0.2–0.9%		[70, 176]
Skeletal muscle	1	Illumina EPIC	282	20.6%		[83]

^a^We account for the different association analyses, even if they are published in the same paper

^b^If more than one analyses is available, the range is presented

#### Tissue-shared meQTLs-CpGs

The majority of DNA methylation signatures are tissue-specific and reflect the developmental trajectories of each cell line [13]. However, when DNA methylation levels are partially or fully driven by genetic variants, DNA methylation levels and meQTLs effects can be tissue-specific or they can also be shared across tissues. Several studies have explored this question, focusing on how easily accessible tissues such as blood may be used as proxies for the indirect study of difficult-to-reach tissues. In a report including samples from T cells, temporal cortex, neurons, glia, and placenta profiled with the 450K array, Do et al. [70] found good overlap in the percentage of meQTL-associated CpGs between temporal cortex with those in neurons/glia (61%) as expected, but not with T cells (28%) or placenta (12%). However, the study explored in a small to moderate sample size (*n*≤54 for each sample type), and consequently had limited power for detection of modest effects and their tissue-specificity assessment. Lin et al. [73] explored meQTLs in 197 saliva samples from control and schizophrenia/schizoaffective disorder patients and compared their results with two previous studies in brain and blood samples. They estimated that 38–73% of the meQTL variants in each tissue are shared with another and that most have a consistent effect direction across tissues. They found that 31–68% of the significant CpGs harboring meQTLs in a certain tissue are also significant in at least one other tissue. From these results, the tissues that share most meQTLs or most CpGs with meQTLs—with at least one other tissue—were blood and saliva. Another interesting observation was that tissue-shared signals were enriched in genetic risk loci of diseases such as schizophrenia, as well as in cross-tissue eQTLs (i.e., eQTLs significant in both blood and brain tissue). Similarly, Qi et al. [84] assessed the correlation of genetic effects at the peak *cis*-meQTLs in blood and brain from five data sets profiled on the Illumina 450K array. The correlation of meQTL effects between two sets of samples profiled in the same tissue was strong (correlation coefficient $\hat {r_{b}} = 0.92$ for both blood and brain sample types), and lower, although still considerable, between brain and blood samples ($\hat {r_{b}} = 0.78$). Other cross-tissue meQTL analyses have also included comparisons between blood, brain, adipose tissue, breast, kidney, and lung samples [50, 56, 65, 85, 86].

Although no clear consensus currently exists in the estimated proportion of tissue-shared meQTLs, increasing evidence shows that a major subset of meQTL-CpG pairs are indeed shared among multiple tissues and cell types.

### MeQTLs databases

Several efforts have attempted to create databases of meQTL findings. One of the first online repositories that incorporated results from GWAS of DNA methylation was GRASP, where the current build has 52,419 meQTLs records [87, 88]. In 2015, Relton et al. [89] constructed the Accessible Resource for Integrated Epigenomic Studies (ARIES), summarizing findings from DNA methylation analysis of 1018 mother-offspring pairs from the Avon Longitudinal Study of Parents and Children (ALSPAC). The resource also includes one of the few longitudinal meQTL studies to date, complementing the original database [37]. The Brain xQTL Serve is another resource that reports results of genetic variation in three molecular traits—gene expression, DNA methylation, and histone acetylation—from prefrontal cortex samples of two longitudinal aging cohorts [57]. In cancer research, the Pancan-meQTL [72] and DNMIVD (for DNA Methylation Interactive Visualization Database) [90] use data from The Cancer Genome Atlas (TCGA). Pancan-meQTL reports 8028 *cis* and *trans*-meQTLs identified in 7242 samples from 23 different tumor types, while DNMIVD complements the Pancan-meQTL findings with additional analyses, such as diagnostic and prognostic models, and pathway-meQTL. Hannon et al. [38] published an interactive database of meQTLs from a blood-based study in 1111 samples, along with putative pleiotropic associations of meQTLs and multiple traits. Altogether, QTLbase is probably the most comprehensive resource to date in different sample types. It compiles summary statistics for molecular QTLs from 233 studies, with meQTL associations representing 16% of the database and summarizing results from 39 meQTLs publications in different tissue types [91]. In blood specifically, the GoDMC resource [50] includes an online searchable tool with a full list of meQTLs from the largest blood meQTL study to date (see the “MeQTLs in blood-based samples” section).

## Genetic effects on DNA methylation: potential underlying mechanisms

### *Cis*-meQTL mechanisms

Despite the identification of hundreds of thousands of associations between meQTLs and CpGs, the molecular mechanisms underlying meQTLs are not well characterized. The leading hypothesis to explain *cis*-meQTL effects is that SNPs in protein binding sites alter or disrupt the activity of sequence-specific binding proteins—such as transcription factors (TFs)—and change methylation patterns of nearby CpGs, either directly or through a signaling cascade [59, 67, 70, 92, 93]. In support of this hypothesis, Banovich et al. [67] showed that for meQTLs in TF binding sites (TFBSs), different alleles predicted to affect affinity of TF binding were correlated with methylation levels at nearby CpG sites. Wang et al. [75] also showed consistent findings by identifying DNA motifs associated with methylation levels, as previously described (see the “MeQTLs are differentially distributed across the genome” section). The authors profiled binding profiles of 845 TFs and concluded that TFs can interact with DNA motifs that are also associated with DNA methylation levels. These results are also in concordance with mechanisms reported to underlie other DNA regulatory pathways and their QTLs, such as histone modifications and RNA polymerase II [94].

**Fig. 2:**
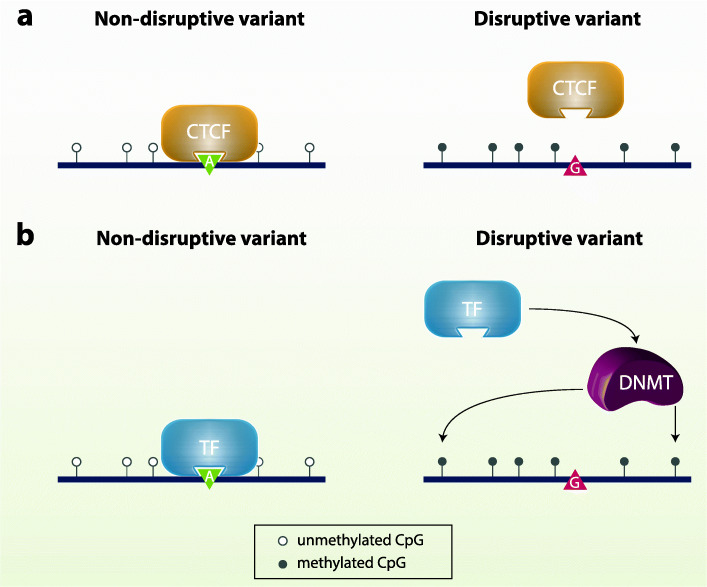
Mechanisms underlying *cis*-meQTL effects. **a** Passive mechanism. Under normal conditions a sequence-specific binding protein (such as CTCF) can bind to its target and prevent methylation changes at surrounding CpG sites due to its occupancy. If a meQTL disrupts the site, the protein cannot bind successfully, and the CpG sites are prone to change in baseline methylation status. **b** Active mechanism. If a meQTL is located in a TFBS, lack of TF binding can promote the recruitment of DNMT or TET enzymes, and thus modify the methylation status of nearby CpG sites

The signaling pathways triggered by sequence-specific binding proteins are still under discussion, but the main premise is that if a TFBS is occupied, this could be enough to prevent DNA methylation changes in the vicinity of this TFBS. This would represent a form of passive control of genetic variation on DNA methylation, via TFBS occupancy (Fig. 2a). Alternatively, TFs could recruit DNMT3A and TET enzymes for active methylation or demethylation (Fig. 2b). This is supported by the observation of an overlap of TFBS with methylation-associated DNA sequence motifs [75].

One of the main examples in support of the hypothesis of passive genetic control on DNA methylation is CTCF (CCCTC-binding factor), which is an insulator involved in chromatin regulation, forming loops and bringing together genetic elements that may be physically far apart. CTCF binding sites usually contain CGI motifs and have to be poorly methylated to allow for the recruitment of the protein [95]. The occurrence of a meQTL within the CTCF binding site may result in a decrease or even annulment of CTCF binding affinity, which in turn can lead to an increase in DNA methylation of nearby CpG sites, as shown in the mouse methylome [96]. Multiple studies have now highlighted CTCF binding as a key example of *cis* genetic-epigenetic interactions [56, 70, 71, 79, 97].

An example of *cis*-meQTL active mechanisms involves a genetic variant within the gene underlying a clinical subgroup of colorectal cancer known as MSI+ (or microsatellite-unstable cancer). Here, decrease of gene expression of the DNA mismatch repair gene MutL homolog 1, *MLH1*, is due to hypermethylation of its promoter. The A allele of variant rs1800734 in the 5 ^′^UTR of *MLH1* modifies the binding of TFAP4 activating the BRAF/MAFG pathway, which increases DNMT3B-mediated methylation of the *MLH1* promoter [98]. Another example of active genetic-methylation interplay is a mechanism suggested to underlie a type 2 diabetes (T2D) susceptibility locus [99]. The T allele of rs11257655 in the *CAMK1D* gene decreases DNA methylation in *CAMK1D* promoter as a meQTL, increases *CAMK1D* expression as an eQTL, and increases T2D risk as T2D GWAS signal. The authors propose that in the presence of the T allele at rs11257655, a protein complex formed by FOXA1/FOXA2 and other TFs binds to an enhancer of *CAMK1D*, which leads to demethylation of cg03575602 in the *CAMK1D* promoter and in turn upregulates its expression.

### *Trans*-meQTL mechanisms

**Fig. 3:**
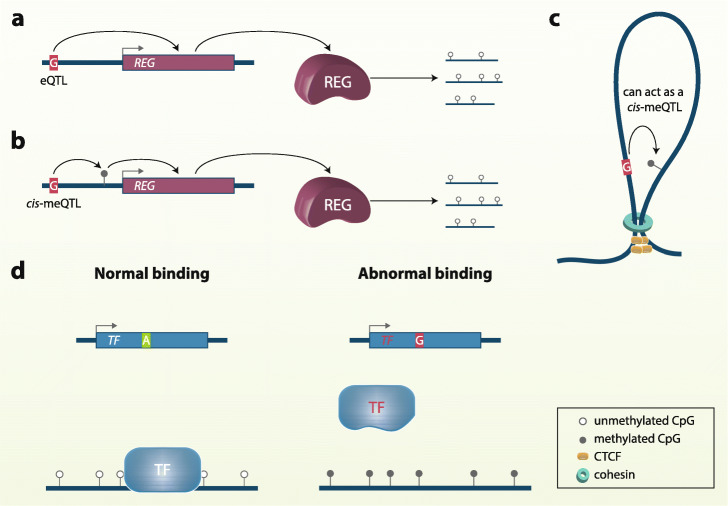
Mechanisms underlying *trans*-meQLTs effects. **a** eQTL-mediated mechanism. If a SNP acts as an eQTL for a gene that regulates DNA methylation, the SNP can have an indirect effect on multiple CpG sites in *trans*. **b***Cis*-meQTL-mediated mechanism. If a SNP is a *cis*-meQTL for nearby CpG sites, which in turn impact the expression of genes involved in epigenetic regulatory processes, the SNP can ultimately alter DNA methylation levels at CpG sites in *trans*. **c** 3D organization mechanism. In the 3D genome, distal sites can move in close proximity, whereby a SNP can affect a DNA methylation levels at CpG sites in *trans*, acting either through *cis*-meQTL mechanisms, or by disrupting the formation of structural loops. **d** SNPs in the coding regions of methyl-specific binding proteins (such as MeCP2) can alter their specificity and function, and therefore passively or actively (by recruiting DNMTs or TETs) modify DNA methylation of their binding sites

Many mechanisms have been hypothesized to underlie *trans*-acting meQTLs effects, but to date, very few clear examples have been uncovered. The simplest hypothesis is that SNPs that act as eQTLs of global methylation regulators, or their associated elements, have downstream effects as meQTLs at multiple CpG sites genome-wide (Fig. 3a). For example, Lemire et al. [55] documented the case of SUMO-specific protease 7 (SENP7), which interacts with epigenetic repression proteins. Intronic variants located in *SENP7* gene are *cis*-eQTLs, and high levels of the transcript decrease methylation at several *trans*-CpGs. Another case is variant rs12933229 associated with expression of *RRN3P2*, a pseudogene that regulates DNA methylation through piwi-interacting RNAs (piRNAs).

Other findings suggest that distal effects may be mediated, in total or in part, by *cis*-meQTL-associated CpGs (Fig. 3b). For instance, one-third of the 585 *trans*-meQTL-CpG pairs identified by Shi et al. [56] in lung tissue showed weaker associations after conditional analyses, conditioning on the *cis*-regulated CpGs by the same SNPs. In 166 *trans*-meQTL associations, the authors found a partial mediation of *cis* effects, with lower but still significant partial correlations compared to marginal correlations, and in 30 associations, they found a full mediation, with no significant correlations after conditioning for *cis*-meQTLs. Genes for GTPase or related enzymes involved in DNA methylation regulation, were over-represented for such *cis*-CpGs. Therefore, one potential mechanism underlying *trans*-meQTL effects is that a meQTL may act on nearby CpGs, which then impact the expression of genes that eventually may modify DNA methylation levels at distal sites.

Three-dimensional (3D) genome conformation changes would be an alternative track for the action of *trans*-meQTLs, since distal loci can be brought into physical proximity by 3D structures [100]. Hence, either SNPs in TFBSs acting as *cis*-meQTLs, or SNPs in sites that anchor cohesins and CTCF that integrate topologically associating domains (TADs) and loops, could have an impact on remote CpGs as they move in closer proximity in complex 3D DNA structures (Fig. 3c) [26]. Furthermore, the 3D organization of the DNA includes inter-chromosomal contact, which would be the source of a fraction of meQTLs associations—as demonstrated by high-resolution Hi-C data that CpGs overlap with binding sites of architectural proteins (e.g., CTCF, RAD21, and SMC3) [51], and with a two-dimensional functional enrichment [50].

Other explanations involve sequence-specific binding proteins, similar to mechanisms for *cis*-meQTLs, but instead of the genetic variant being located in TFBS, here, the SNPs interfere with the coding or *cis*-regulatory regions of the TFs, and thus their subsequent expression, coupling, and function (Fig. 3d). The results of Bonder et al. [51] point in this direction. The authors found that 13.1% of the *trans*-meQTLs that they detected also altered the expression of TFs, and those affecting multiple CpGs had consistent direction of effects, either increasing or decreasing methylation at most of CpGs. A representative example is rs3774937 in the intron of the TF *NFKB1*, which is a *trans*-meQTL for 413 CpG sites genome-wide. In 380 CpG sites, the rs3774937 alternative allele was associated with lower methylation levels, and 147 of those CpG sites were in NF- *κ*B binding sites.

The same mechanism could also apply to the activity of proteins other than sequence-specific binding proteins, although this theory remains mostly unexplored so far. For example, it is well known that DNA binding of some proteins is methylation-dependent through a methyl-CpG-binding domain (MBD), such as for the MeCP2 (methyl-CpG-binding protein 2). MeCP2 regulates DNMT3A allosterically, acting as a repressor or an activator of the methylation process [101]. However, some mutations in the *MeCP2* gene decrease selectivity of the MeCP2 binding [102], and consequently, could lead to untargeted methylation at several distal sites. This idea can also be extended to proteins without MBD, as emerging evidence suggests [103]. This may also complement the sequence-specific binding sites theory and thus explain more *trans*-meQTLs.

### MeQTLs and mechanisms underlying human disease

Many research efforts have linked meQTLs to genetic variation underlying human complex traits. MeQTLs are significantly enriched for GWAS signals, with evidence for shared genetic effects [50]. Multiple studies have explored the directionality of shared genetic associations, applying causal inference approaches typically exploring the potential role of DNA methylation as a mediator of genetic effects on phenotypes [38, 50, 104]. However, despite a substantial sharing of genetic effects, the findings reveal a more complex genetic architecture including putative evidence for both mediation effects of DNA methylation on phenotypes, as well as effects of complex traits on methylation (see the “MeQTLs and GWAS” section).

Nevertheless, the discovery of meQTLs has contributed to the advancement of our understanding of the molecular pathways underlying certain human phenotypic traits and diseases, which may eventually help towards the development of therapeutic targets. Examples include a thorough investigation of previously identified genetic signals for Alzheimer’s disease involving the promoter region of gene *PM20D1* and meQTLs (rs708727–rs960603 haplotype) [105]. With a series of in silico, in vitro, and in vivo experiments, Sanchez-Mut et al. determined that meQTLs interact with the promoter of *PM20D1* through haplotype-dependent 3D chromatin conformations via CTCF, changing DNA methylation levels, altering gene expression, and ultimately protecting or aggravating neurodegeneration. In another study focused on characterizing osteoarthritis risk variants in cartilage samples, Rice et al. [106] found four meQTLs for 17 CpGs. In vitro studies of the prioritized locus suggest potential DNA methylation and gene expression mechanisms altering the function of the *PLEC* and *GRINA* genes, which have not been previously described in context of osteoarthritis. Similarly, meQTLs have helped to elucidate biological pathways underlying other diseases such as Parkinson’s disease [107], multiple sclerosis [108], colorectal cancer [98], and T2D [99] (see the “*Cis*-meQTL mechanisms” section), along with complex phenotypes such as platelet function [109], fatty acid levels [110], and others.

## Challenges and future directions

### Methodological and statistical caveats

#### DNA methylation profiling

The vast majority of meQTL studies to date explore DNA methylation levels profiled by Illumina DNA methylation arrays, which are relatively low-cost and highly standardized. However, array-based DNA methylation profiles can be subject to bias introduced by errors from cross-hybridization events, as well as batch and positional effects. For example, positional effects have been reported to impact a larger proportion of 450K probes, compared to 27K probes [111]. In addition, both the 450K and EPIC arrays contain two different types of probes with different dynamic ranges [112]. Several methods have been developed to minimize bias introduced by these potential array effects [113–116], as well as comparisons across methods, which provide useful frameworks for the design of quality control and normalization of Illumina-based DNA methylation profiles [117–119]. Further work has also focused on guidelines for exclusion criteria of low-performing probes [120–122], or has explicitly flagged unreliable probes due to cross-reactive events or underlying genetic variation [123].

As previously discussed, genome coverage is a key consideration in DNA methylation profiling technologies, and here the ultimate aim is to characterize meQTLs across the entire methylome. With most studies based on array DNA methylation profiles, the EPIC array provides a reasonable cost-coverage balance with increased coverage of regulatory elements compared to the 450K. Despite the improvement in coverage by the EPIC array, regulatory regions included on the EPIC only comprise 27% of *cis* and 7% of *trans* regions characterised by ENCODE [123]. This, combined with the limited methylome coverage, should also be considered when generalizing meQTL findings to whole genome.

On the other hand, WGBS allows for comprehensive profiling of the methylome, but the high costs are still restrictive and prevent its broad application in meQTL studies. Also, some genomic regions and difficult to sequence and library preparation protocols are technically complex and may be subject to bias from multiple sources, such as bisulfite conversion, PCR amplification, DNA modifications, and degradation [124, 125]. An important parameter to define in a WGBS experiment is the sequencing depth. The recommended depth coverage based on data from the NIH Roadmap Epigenomics Project [126] and the International Human Epigenome Consortium (IHEC) [127] is 30×. In order to optimize costs while maintaining acceptable rates of specificity and sensitivity, Ziller et al. [128] proposed a minimum coverage per sample of 5–15 × for the discovery of differentially methylated regions (DMRs). Nonetheless, coverage of 100× would be required to have similar precision to that in Illumina arrays [124]. In light of these estimates, WGBS in large-scale samples currently still poses significant challenges, but represents a promising method for future meQTL analyses, especially for studying regions of the genome underrepresented in microarrays.

#### Statistical models

The choice of statistical model for meQTL analysis is important. Most meQTL studies apply linear regression, but at many CpG sites the distribution of DNA methylation values does not meet its assumptions, which may in turn increase the error rate (both type I and II). Recently, Mansell et al. [129] quantified the extent of bias in epigenome-wide association studies (EWAS) using the EPIC array due to non-linearity between variables, non-normal distribution of residuals (skewness and kurtosis), and heteroskedasticity. The authors concluded that even CpG sites with extreme deviation to linear regression assumptions do not result in major bias. By extension, this observation could also apply to meQTL studies. Interestingly, the same study did not find better performance when using *M*-values instead of *β*-values in DNA methylation analysis. Ultimately, a higher selectivity of the CpG sites to test—such as filtering out probes with low *β*-values variability—would leverage the statistical confidence of the models and maximize reproducibility of results, as recommended by Logue et al. [122].

#### Multiple-testing correction

One major consideration in meQTL analyses is the multiple-testing correction, given the large number of tests in comparing millions of genetic variants against typically at least hundreds of thousands of CpG sites. On the one hand, the multiple testing correction must be computationally efficient, and on the other hand, the aim is to maximize statistical power to detect low or modest effects.

A considerable amount of studies apply permutation-based multiple testing thresholds to quantify an empirical false discovery rate (FDR) [130]. Typically, this approach consists of randomizing the genotypes to generate an approximate null distribution of *p*-values obtained in a large number of association tests between the permuted genotypes and CpG sites. The FDR is the ratio of associations in the permuted data to those observed at a specific nominal significance threshold [131]. Because permutations are computationally demanding, methods as FastQTL and QTLtools [132, 133] have proposed variations of the original technique, such as drawing a few thousand permutations and modeling the resulting *p*-values with a beta distribution to approximate the null distribution. This approach has been adopted by some meQTLs analyses [83, 93]. Moreover, some analyses have reduced the number of permutations, for example to a hundred [60] or even ten [51, 82], a decision supported by eQTL results about the stability of the FDR value with as few as five permutations [134, 135]. For example, *cis*-meQTL analysis by van Dongen et al. [39] in buccal cells of MZ twins applied the aforementioned method with ten permutations, conserving relatedness between twins by permuting twin pairs samples rather than individuals.

Other studies have applied the conservative Bonferroni multiple testing correction to control the family-wise error rate (FWER), adjusting for the total number of SNPs-CpGs pairs tested, resulting in stringent multiple testing significant thresholds (e.g., *p*<1×10^−10^) [35, 37, 136]. However, the Bonferroni multiple testing correction does not take into account linkage disequilibrium (LD) between genetic variants or patterns of co-methylation, that is, the correlation in DNA methylation levels at nearby CpG sites [137]. To tackle LD, McRae et al. [53] and Hannon et al. [38] employed a Bonferroni threshold based on the GWAS canonical value 5×10^−8^—which accounts for LD blocks—and divided this threshold by the number of tested probes, while Smith et al. [86] adopted a Holm-Bonferroni method—or a step-down Bonferroni—which increases the power. To take into account co-methylation, it has been proposed that for the Illumina EPIC array in whole blood, an appropriate choice of the number of independent probes to control the FWER would be 530,639 (66% of total sites) [129].

MeQTL analysis strategies that use the Bayesian framework [138] or a multivariate normal distribution [139] have also been applied to other molecular QTL studies, and appear promising to explore in future meQTLs analyses.

### Detection of common and rare variant meQTLs

Since publication of the first genome-wide meQTL studies, sample sizes have increased dramatically and with them power to detect small effects of common genetic variants on methylation. However, the detection of rare genetic variants is still a major challenge in meQTL studies. Almost all genome-wide meQTL studies discard SNPs with a minor allele frequency (MAF) less than 0.05 or 0.01, while the high penetrance of rare variants in certain complex traits highlights their biological importance [140].

The most widely implemented approaches for assessing effects of rare genetic variants on human complex traits are collapsing methods. The premise is that all the variants within the boundaries of a functionally meaningful locus would induce the same phenotypic change [140]. To our knowledge, only the study by Richardson et al. [141] has so far examined rare variants in meQTL analysis in blood samples. The authors collapsed variants with MAF ≤0.05 around CGIs (alone and with flanking shores/shelves) and carried out the Sequence Kernes Association Test (SKAT) testing for genetic influences on CpG sites from the 450K array. The results identified 94 unique *cis*-acting and one *trans*-acting regions, which were not previously linked to methylation. This novel approach can be leveraged in future meQTL analyses by the definition of other functional units for collapsing regions, testing previously identified meQTL regions after conditional analysis, and application to data from the EPIC array or WGBS.

### Gene–environment interactions

Environmental exposures can leave a clear signature on DNA methylation patterns, as observed for smoking [18, 142, 143] and alcohol consumption [19]. Some environmental exposure or lifestyle factors and behaviors also have a genetic component that explains a proportion of their variance [144, 145]. Therefore, to explore the interplay between genetic variation, environmental exposures, and epigenetic changes, some studies have considered gene–environment (G ×E) interaction terms in meQTL analyses.

To date and to our knowledge, no genome-wide G ×E analysis of DNA methylation has yet been published. However, G ×E analyses at candidate genomic regions have been described in several studies. For example, Teh et al. [146] studied the interaction of genetic effects and in utero environment in 237 umbilical cord samples in Asian neonates. Firstly, the authors identified 1423 variably methylated regions (VMRs) across individuals, based on the median absolute deviation of the DNA methylation levels in each CpG site. Then, to explore triggers of DNA methylation changes at each VMR, the authors assessed DNA methylation effects as a function of (1) genotype alone, (2) intra-uterine environment alone, or (3) the G ×E interaction. The intra-uterine environment was quantified through 19 parameters, including maternal smoking, maternal depression, and concentrations of compounds in maternal serum. Interaction models of genotype with different in utero environments had better performance at 75% of the VMRs compared to main-effects models, and therefore better explained the variability in DNA methylation. In two other studies [60, 147] of monocyte samples from European and African populations, the authors suggested that some of the meQTLs may in fact be occurrences of G ×G and G ×E interactions (see the “MeQTLs are differentially distributed across the genome” section). The *cis* analysis uncovered 69,702 CpGs with meQTLs, and of these, 4.1% displayed different effects across the two populations, which may reflect G ×G or G ×E interactions.

Several studies have explored G ×E effects involving smoking status and genetic variants at candidate loci in the context of meQTLs and complex disease. Meng et al. [148] provide an example of a candidate G ×E effects linked to rheumatoid arthritis, involving genetic variants in the MHC and smoking status. They observed an effect of rs6933349 on cg21325723 (located in the body of the *TSBP1* gene), only in current smokers. Further examples include the study of Klengel et al. [149] who investigated a G ×E interaction in *FKBP5*, a gene that regulates the glucocorticoid receptor—a major component of the stress hormone system. The transcriptional activation of *FKBP5* as a response to childhood abuse depends on genetic variants (rs1360780) that alter the 3D conformations of the locus; the expression of the gene is mediated by the demethylation in intron 7, a change that is long-term stable and has implications in stress disorders. A similar pathway potentially underlies methylation at *SLC6A4* (serotonin transporter gene) [150].

The implementation of G ×E meQTL analyses entails challenges, such as substantially larger multiple testing burden and limited power [151]; however, it represents a promising niche to explore that may account for a fraction of missing heritability in DNA methylation.

### MeQTL impacts on DNA methylation variance

Conventional statistical methods applied to QTL analysis aim to identify significant deviations of the trait mean between the subjects in different genotype groups, typically with the assumption of equal variance across groups (i.e., homoskedasticity). Recently, new perspectives in QTL studies have explored QTLs that influence the phenotypic variability across genotype groups, or variance QTLs (vQTLs/varQTLs). VarQTL may capture interaction effects, such as epistatic (G ×G) or G ×E [152]. Methods for detecting varQTLs include parametric and non-parametric tests, including Bayesian and family-based approaches [152–154]. One of the most comprehensive studies on varQTLs was carried out recently by Wang et al. [155], with genotype data from 348,501 participants from the UK Biobank and across 13 quantitative traits—including obesity-related, height, and lung function measures. The results show a total of 75 varQTLs for nine traits (54 varQTLs related to obesity) located in 41 nearly independent loci. Moreover, the authors found two varQTLs with possible non-additive effects on the variance, 66 varQTLs that also have an effect on the mean of the trait with the same direction, and 16 varQTLs that are explained by G ×E interaction models.

So far, only few studies have explored varQTLs in the context of molecular datasets, such as DNA methylation or gene expression profiles. For example, Brown et al. [156] examined variability of 13,660 genes in 765 LCL samples from the TwinsUK cohort, identifying 508 var-eQTLs in *cis*, of which 36% were also eQTLs. They then searched for variants interacting with each of the var-eQTLs within the same *cis* window in order to identify epistatic interactions, and found 256 G ×G signals, of which 57 replicated in another cohort. They also suggested that 70% of var-eQTLs may be the result of G ×E interactions based on analyses focusing on gene expression differences in MZ twins. In a methylome analysis with the 450K array in 729 peripheral blood leukocytes samples from individuals of Swedish descent, Ek et al. [157] estimated a total of 374,252 CpG-var-meQTL pairs, or 7195 unique CpGs with at least one var-*cis*-meQTLs. At almost all of these CpGs, there was also evidence of *cis*-meQTL effects, and after adjusting methylation levels for *cis*-meQTLs, the authors no longer found variance heterogeneity at the majority of CpGs. As a result, they conclude that a considerable proportion of varQTLs (92%) may be statistical artifacts attributed to SNPs in LD, rather than real biological interactions, and that var-meQTLs are unlikely to explain missing heritability.

Future studies are needed to replicate these var-meQTLs results, explore mechanisms driving these effects, and potentially identify novel signals.

### Integrating meQTL results in association studies

#### MeQTLs and EWAS

EWASs aim to systematically associate variation in DNA methylation levels across the genome with variation in phenotypes or environmental exposures. However, significant associations between DNA methylation levels and phenotypes may arise due to confounding effects of meQTLs, and most EWASs do not take meQTLs into account. Adjustment of DNA methylation values for meQTL effects prior to EWAS has been proposed to tackle this issue [158, 159]. Chen et al. [78] applied this approach in EWASs of gene expression levels genome-wide, or in expression quantitative trait methylation (eQTM) analyses. The authors quantified the contribution of DNA methylation to gene expression variance through a variance decomposition model and found that DNA methylation explained a lower proportion of the variance in models adjusted for underlying genetic effects, compared to unadjusted models. Subsequently, they performed EWASs with two models—either not correcting for or correcting for *cis*-genetic effects. Over half of the genes associated with epigenetic marks in the uncorrected model did not reach significance in the corrected model. Although meQTLs effects were not directly assessed in this study, these findings may extend to meQTLs. In another study, Krause et al. [160] aimed to validate two candidate CpGs associated with T2D, but found a significant association between BMI and blood methylation only after correcting for genotype at rs9982016, which was a *cis*-meQTL at one of the candidate CpGs.

Another relevant application of integrating meQTLs in EWAS is to gain insight into the putative causal direction of association between DNA methylation signals and the associated phenotypes by using Mendelian randomization (MR). MR evaluates the likelihood that a phenotype is the consequence of an exposure, which in turn is the result of genetic variation (or the instrumental variable) [161]. In context of epigenetic analyses, meQTLs are instrumental variables, DNA methylation levels are exposures, and diseases or phenotypes are the outcomes [104]. Multiple studies have applied MR using meQTLs in EWAS across a range of phenotypes [162–166]. For example, in an EWAS of BMI in 3743 blood 450K methylomes from older adults and with replication, Mendelson et al. [167] identified 83 DMSs and their associated meQTLs. Follow on MR identified two CpGs (cg11024682 in *SREBF1*, and cg07730360, unannotated) with nominally significant putative causal effects of DNA methylation on BMI. In contrast, they identified 16 CpG where DNA methylation levels are likely mediated by BMI after a reverse MR model. Using a similar approach, Dekkers et al. [168] analyzed if exposure to elevated blood lipids affected DNA methylation levels in immune cells, in 3296 450K methylomes from six Dutch biobanks. The authors identified 21 DMSs for triglycerides (TG) levels, three for low-density lipoprotein cholesterol (LDL-C) and four for high-density lipoprotein cholesterol (HDL-C). Follow on MR analysis identified putative causal effects of lipid levels on 13 DMSs. To exclude pleiotropy (SNPs acting as QTLs for multiple lipid levels, or as *cis*-meQTLs in DMSs) and reverse causation (*cis*-meQTLs affecting DMSs, and DMSs affecting lipid levels), the authors conducted secondary MR analysis. The results confirmed that TG likely induced differential methylation at three CpGs, LDL-C at one, and either TG or HDL-C at two. Mendelian randomization has also been applied when integrating meQTL results and GWAS (see the “MeQTLs and GWAS” section).

In addition, the combination of EWAS and meQTL signals can be used to explore G ×E interactions. For instance, Tsaprouni et al. [169] found that almost half of smoking-associated loci have meQTLs. Subsequent analyses fitting G ×E interaction effects identified a CpG (cg03329539 located in chromosome 2) where methylation response to cigarette smoking was modulated by rs62192178 genotype.

#### MeQTLs and GWAS

Although thousands of GWAS results have been published to date, the identification of causal variants and their functional interpretation remains mostly outstanding. Furthermore, GWASs also face the “missing heritability” problem and epigenetic signals (potentially, through meQTLs) might explain a proportion of the phenotype missing heritability [27, 170]. Therefore, integrating meQTL findings as a post-GWAS analysis can help to address some of these challenges.

One approach for this integration is to use meQTLs findings to prioritize GWAS signals for follow on analysis, for example, as applied in a study of autism spectrum disorders in 1263 infants by Hannon et al. [171]. The authors estimated that 91 SNPs associated with the disease were also meQTLs, based on a Bayesian co-localization analysis. Their results highlight specific variants to target in subsequent studies since they may have a functional role in autism pathophysiology. Morrow et al. [82] implemented a similar Bayesian framework to identify meQTLs that are also chronic obstructive pulmonary disease (COPD) GWAS signals (see the “MeQTLs in non-blood-based tissues and cells” section). Their findings identified 20 SNPs with suggestive evidence of co-localization, highlighting novel regions of interest in addition to previously identified COPD signals, such as *KCNK3* and *EEFSEC*.

MR analyses have also been adopted to integrate meQTL and GWAS results. Richardson et al. [172] assessed putative causal effects at 30,328 CpGs in 139 complex traits based on previously published *cis*-meQTL and GWAS results. The authors assessed the fit of several models spanning: (1) a forward MR model where the DNA methylation level impacts the phenotype; (2) a joint likelihood mapping, to exclude genetic variants in LD independently influencing DNA methylation and phenotype; and (3) a reverse MR model to exclude cases where DNA methylation is the outcome. A final set of 346 CpG sites were identified as potentially causal across 46 traits, ultimately highlighting specific biological pathways and suggesting potential drug targets. Similar analyses have also been undertaken within specific phenotype domains by multiple other studies, including Huan et al. [35], Bonder et al. [51] and Chen et al. [78]. In the largest analysis so far, Min et al. [50] found a significant substantial enrichment of meQTLs with the GWAS signals in 13 of 37 phenotypes GWAS datasets assessed, especially for SNPs acting as both *cis* and *trans*-meQTLs. However, after multiple causal inference analyses, the authors observed that only for a minority of cases DNA methylation exhibited mediating effects of GWAS signals in complex traits, and vice versa. These directionality results have several interpretations, including the possibility that other molecular mechanisms may explain a proportion of the observed shared genetic signals.

#### Shared QTL effects on multiple regulatory genomic processes

Regulatory genomic changes capture multiple molecular processes across different layers of epigenetic data. Comparison of meQTLs with QTLs for different biological profiles is a promising route to infer regulatory potential. In spite of the considerable amount of studies that jointly consider DNA methylation and gene expression data, relatively few studies have explicitly compared eQTLs and meQTLs genome-wide. Such comparisons have been based on either summary statistics of published studies [92, 173] or de novo associations [49, 51, 59, 67, 78, 83, 93]. Overlapping results can be used to identify pleiotropic effects for DNA methylation and expression and explore directionality of these effects, such as SNP →methylation →expression (active) or SNP →expression →methylation (passive). For example, Gutierrez-Arcelus et al. [59] inferred that DNA methylation can have both active and passive roles in gene expression regulation across fibroblasts, T cells and lymphoblastoid cells from the umbilical cords of 204 babies. Furthermore, comparison of meQTLs with other epigenetic data QTLs may also give further insights into regulatory epigenetic processes. Banovich et al. [67] compared meQTLs with QTLs for histone modifications, PolII occupancy and DNAse I hypersensitivity, and based on the extent of overlap observed they hypothesized that coordinated regulatory changes may be explained by modified TF binding affinities. Chen et al. [78] explored similar questions in three different immune cell types (*n*=525), where 43.3% of the genetic variants identified as eQTLs were either found to have a coordinated effect as meQTLs or to be in high LD with a meQTL. However, the effect sizes were weakly negatively correlated, which the authors interpreted as a partial uncoupling between methylation and expression. The study also included analysis of histone modification QTLs (hQTLs for H3K4me1 and H3K27ac), where again 43.3% of eQTLs and hQTLs overlapped with strong positive correlation in effect sizes, suggesting an active role for histone modifications on expression.

As additional data are being generated on multiple epigenetic and expression layers of data, future analyses will have greater power to explore the regulatory nature of meQTLs. However, co-localization results should be interpreted with caution, as the intersection of QTLs does not imply a causal relationship or direct association due to LD or statistical artifacts. Additionally, if summary statistics are obtained from databases with different reference populations, the significant signals may not be comparable [25].

In conclusion, the identification of methylation quantitative trait loci genome-wide has significantly increased our knowledge of the factors driving DNA methylation variation in humans, and holds value for integrating genomics and epigenomics in the context of disease.

## Supplementary Information

**Additional file 1** Review history
