# Supplementary material for: Genetic impacts on DNA methylation: research findings and future perspectives
Source: Genome Biol. 2021 Apr 30;22:127. doi: 10.1186/s13059-021-02347-6 (PMC8086086; doi:10.1186/s13059-021-02347-6)
Supplement: Supplementary file 1 — Additional file 1 Review history [file 13059_2021_2347_MOESM1_ESM.docx]

Review History

**First round of review**

**Reviewer 1**

**Comments to author:**

Villicana et al presents a very detailed overview of current findings from genetics studies of DNA methylation focusing mainly about 1) methylation measured by targeted arrays and 2) SNP vs CpG methylation (QTL) association. It is well written and covers relevant studies in the field. However, as the review is limited to these two aspects there are too much details presented that is irrelevant for the reader. For instance, the authors write "Published thresholds are typically of the order of p < 1x10-5 for cis effects and p < 1x10-9 for trans …" Instead, highlighting effect sizes identified across studies would be preferred. The addition of these too many details is not causing the review to be very lengthy but also hinder the reader to summarize and conclude relevant findings.

Another concern is the structure of the review where a subject or study is discussed multiple times in different sections which result in not only overlap but confusion as the conclusions are often drawn at the latter sections. The suggestions would be to rearrange (and reduce) the text to avoid this either by removing some of the details in the first half of the review or combine sections. Here are some examples:

1. DNA methylation heritability studies are discussed on page 2 where the authors conclude that various studies show relatively low h2 (~0.2) without further discussions about the limitations of targeted arrays for assessing genome wide estimates. Instead this is brought up in the section "Future perspectives" on page 13.

2. On page 8, the authors bring up the concept of Mendelian Randomization analysis highlighting multiple studies applying this approach without further details or description of the rationale for applying this approach. However, on page 17 (again under the "Future Perspective") they describe this in detail.

3. Similarly, on page 8, they briefly discuss examples of coordinated effects of genetic variants citing Chen et al which is further described in the "Future Perspective" section.

The review would appear more appealing if it would go beyond the current narrow focus. For instance, allele-specific methylation is only briefly mentioned. Recent studies applying WGBS include PMID: 33067439, PMID: 32594908, PMID: 32460014. Outlining the conceptual difference between meQTL and ASM analysis would add value along with highlighting key findings from recent ASM studies.

**Reviewer 2**

**Comments to author:**

The authors present a thorough review of an important but rather disparate field that is certainly in need of some good reviews that help tie together the efforts of the last decade. The review is well written and informative, but could can be improved in several aspects.

For reviews such as this, which are cross-disciplinary, it is vital that they are written in such a way as to carry readers from the each discipline (genetics, epigenetics, population genetics, bioinformatics, EWAS, GWAS, etc) through the article. As an epigeneticist with some (albeit minor) experience of EWAS, I found the review tough-going in some parts, but having chewed through it found it a highly valuable, interesting and enlightening piece. Thus, many of my suggestions relate to accessibility:

1. Figures and information boxes are lacking. Far more figures should be included in such a review. For example, a figure showing how methylation arrays (the major technology in the field) work (and their limitations, … snps, distribution of probes, etc) is very important, as is a figure illustrating the main concepts in heritability

2. Each section should start with a couple of sentences to lead in the reader. For example, the section entitled 'DNA methylation heritability' begins with a rather heavy sentence stating technical definitions of heritability. Instead, the authors should starting each section in the review with a couple of guiding sentences that explain why the topic of each section is so important …. 'key to the principle of meQTLs is that DNA methylation variation is heritable … to establish the heritability of DNA methylation in humans researchers use ……'

3. The authors should make more of an effort to reference original articles throughout. Several sections (i.e. Introduction) heavily cite other reviews, which can be appropriate, but I felt was a little too much

4. 'meQTLs are differentially distributed across the genome' - simple figure here would be good here

5. Page 4, lines 16-28 are important (lack of intergenerational epigenetic inheritance) and should be highlighted - maybe its own subheading - probably showing my bias here

6. A 'Box' defining the terms (which are many and from many different disciplines) used in the review is required

7. Finally, meQTLs have been studied for a while now in the context of many diseases; have any of these studies really (clearly and reproducibly) lead to advancement of our understanding of any human disease or phenotype (prognosis, prediction, treatments)? If so, what? The authors make a statement to this effect in the conclusion, but I am unclear if this is true. Would be nice to see some concrete meQTL data that lead to a real advancement in understanding if such evidence exists (excluding interesting associations etc.).

8. The Discussion is very detailed and well-written, but the conclusion is very vague and doesn't add anything. Is it required? Maybe it can be reduced to a single sentence at the end of the discussion.

**Authors Response**

**Point-by-point responses to the reviewers’ comments:**

We thank the Reviewers for their careful and thorough comments. We have addressed the comments in a point-by-point response in **bold typeface.**

Reviewer #1: Villicana et al presents a very detailed overview of current findings from genetics studies of DNA methylation focusing mainly about 1) methylation measured by targeted arrays and 2) SNP vs CpG methylation (QTL) association. It is well written and covers relevant studies in the field. However, as the review is limited to these two aspects there are too much details presented that is irrelevant for the reader. For instance, the authors write "Published thresholds are typically of the order of p < 1x10-5 for cis effects and p < 1x10-9 for trans ..." Instead, highlighting effect sizes identified across studies would be preferred. The addition of these too many details is not causing the review to be very lengthy but also hinder the reader to summarize and conclude relevant findings.

**We have restructured the review in line with the Reviewers’ comment below, and have reduced the redundancy and the overall length of the review.**

**We agree that effect sizes are more relevant and descriptive for presenting results of meQTL studies. However, published studies do not systematically report effect sizes, but they always report P-values. We explicitly include this comment in the revised manuscript (page 6, lines 18–21).**

**We have kept the discussion of meQTL significance thresholds, because different criteria can impact genome-wide meQTL estimation and their interpretation.**

Another concern is the structure of the review where a subject or study is discussed multiple times in different sections which result in not only overlap but confusion as the conclusions are often drawn at the latter sections. The suggestions would be to rearrange (and reduce) the text to avoid this either by removing some of the details in the first half of the review or combine sections. Here are some examples:

1. DNA methylation heritability studies are discussed on page 2 where the authors conclude that various studies show relatively low h2 (~0.2) without further discussions about the limitations of targeted arrays for assessing genome wide estimates. Instead this is brought up in the section "Future perspectives" on page 13.

**We agree with the Reviewer. We moved the relevant text on interpretation of heritability results in the section of “DNA methylation heritability” on pages 4–5, and now also cross reference the “Methodological and statistical caveats” section.**

2. On page 8, the authors bring up the concept of Mendelian Randomization analysis highlighting multiple studies applying this approach without further details or description of the rationale for applying this approach. However, on page 17 (again under the "Future Perspective") they describe this in detail.

**We have now removed references to Mendelian randomisation analysis (originally on page 8), and only mention it in the section “Integrating meQTL results in association studies”.**

3. Similarly, on page 8, they briefly discuss examples of coordinated effects of genetic variants citing Chen et al which is further described in the "Future Perspective" section.

**We reduced the summary of Chen et al., removing the description of results of coordinated effects on multiple molecular traits (originally on page 8), and only mention it in the “Future Perspectives” section.**

The review would appear more appealing if it would go beyond the current narrow focus. For instance, allele-specific methylation is only briefly mentioned. Recent studies applying WGBS include PMID: 33067439, PMID: 32594908, PMID: 32460014. Outlining the conceptual difference between meQTL and ASM analysis would add value along with highlighting key findings from recent ASM studies.

**We respectfully disagree that the review currently has a narrow focus.**

**We have a brief paragraph on ASM in this review which we have now slightly expanded to incorporate the suggested references, and to now explicitly comment on the conceptual differences, as suggested by the Reviewer (on page 8, lines 7–10). However, given that ASM represents a specific type of meQTL effect with different statistical and technical challenges, we feel that a more comprehensive description on ASM is beyond the scope of this review.**

**Furthermore, there is a nice recent review which focuses only on *cis* genetic effects and includes ASM** [**(**](https://doi.org/10.1186/s13059-017-1250-y)**https://doi.org/10.1186/s13059-017-1250-y). In contrast, our manuscript covers more general meQTL topics that have not been previously addressed in depth, such as *trans* effects, underlying mechanisms and integrative analyses.**

Reviewer #2: The authors present a thorough review of an important but rather disparate field that is certainly in need of some good reviews that help tie together the efforts of the last decade. The review is well written and informative, but could can be improved in several aspects.

For reviews such as this, which are cross-disciplinary, it is vital that they are written in such a way as to carry readers from the each discipline (genetics, epigenetics, population genetics, bioinformatics, EWAS, GWAS, etc) through the article. As an epigeneticist with some (albeit minor) experience of EWAS, I found the review tough-going in some parts, but having chewed through it found it a highly valuable, interesting and enlightening piece. Thus, many of my suggestions relate to accessibility:

1. Figures and information boxes are lacking. Far more figures should be included in such a review. For example, a figure showing how methylation arrays (the major technology in the field) work (and their limitations, ... snps, distribution of probes, etc) is very important, as is a figure illustrating the main concepts in heritability

**We agree with the Reviewer and have added more display items. First, we now include a new Figure 1 outlining the steps in a typical meQTL study, and this includes a panel on methylation technologies. Second, we now also include a new table in the form of a glossary to clarify terminology used in the text.**

2. Each section should start with a couple of sentences to lead in the reader. For example, the section entitled 'DNA methylation heritability' begins with a rather heavy sentence stating technical definitions of heritability. Instead, the authors should starting each section in the review with a couple of guiding sentences that explain why the topic of each section is so important .... 'key to the principle of meQTLs is that DNA methylation variation is heritable ... to establish the heritability of DNA methylation in humans researchers

use	'

**We agree. We have now included introductory sentences in sections “DNA methylation heritability” (page 3), “Methylation quantitative trait loci” (page 6), and “Tissue-specificity of meQTL effects” (page 11).**

3. The authors should make more of an effort to reference original articles throughout. Several sections (i.e. Introduction) heavily cite other reviews, which can be appropriate, but I felt was a little too much

**This has been updated.**

4. 'meQTLs are differentially distributed across the genome' - simple figure here would be good here

**Although we agree with the Reviewer, the different meQTL studies tend to use different enrichment approaches and annotations, and testing frameworks for significance. This makes it very difficult to directly compare and summarise all results in one figure.**

5. Page 4, lines 16-28 are important (lack of intergenerational epigenetic inheritance) and should be highlighted - maybe its own subheading - probably showing my bias here

**We agree. We have included additional text highlighting that in contrast to findings from model organisms, results from human studies on this topic are negative—on page 5, lines 8–13.**

6. A 'Box' defining the terms (which are many and from many different disciplines) used in the review is required

**We agree and now include a new glossary (Table 1).**

7. Finally, meQTLs have been studied for a while now in the context of many diseases; have any of these studies really (clearly and reproducibly) lead to advancement of our understanding of any human disease or phenotype (prognosis, prediction, treatments)? If so, what? The authors make a statement to this effect in the conclusion, but I am unclear if this is true. Would be nice to see some concrete meQTL data that lead to a real advancement in understanding if such evidence exists (excluding interesting associations etc.).

**We added a new brief section “MeQTLs and mechanisms underlying human disease” to address this comment on pages 18–19. The section includes succinct examples of meQTLs that led to a better characterisation of mechanisms in diseases.**

8. The Discussion is very detailed and well-written, but the conclusion is very vague and doesn't add anything. Is it required? Maybe it can be reduced to a single sentence at the end of the discussion.

**We reduced the conclusion to a single sentence at the end of the discussion as suggested.**

**Second round of review**

**Reviewer 1**

The authors have addressed the concerns and I have no further edits.

**Reviewer 2**

The authors have addressed my comments. The new and updated figures and tables have made the review more accessible to a wider audience as have the changes to the text.

The new section entitled, 'MeQTLs and mechanisms underlying human disease' is also a useful addition. However, were the authors not a little surprised by how little they found! - this is why I asked for this section - no action required, just food for thought.

Accept - no further revisions.
